# LncRNA HOTAIR regulates autophagy and proliferation mechanisms in premature ovarian insufficiency through the miR-148b-3p/ATG14 axis

**DOI:** 10.1038/s41420-024-01811-z

**Published:** 2024-01-24

**Authors:** Chao Luo, Lun Wei, Fei Qian, Le Bo, Shasha Gao, Guangzhao Yang, Caiping Mao

**Affiliations:** Reproductive Medicine Center, The First Affiliated Hospital of Soochow University, Suzhou, 215000 China

**Keywords:** Endocrine reproductive disorders, Prognostic markers

## Abstract

Premature ovarian insufficiency (POI) is a serious disease significantly affecting the physical and mental health of women of reproductive age, not just impacting fertility outcomes. Ovarian damage due to chemotherapy remains a major cause of this condition. Recent studies have indicated the involvement of the long non-coding RNA HOTAIR in the progression of various diseases, showcasing important biological functions, yet its role in POI remains unclear. We conducted microarray dataset analysis and qRT-PCR experiments, demonstrating downregulation of HOTAIR expression in ovarian tissue and granulosa cells. Various functional experiments using plasmids overexpressing HOTAIR confirmed its promotion of cisplatin-induced granulosa cell autophagy and proliferation. Mechanistically, dual-luciferase assays showed that HOTAIR modulates ATG14 levels in POI by binding miR-148b-3p, thereby enhancing levels of autophagy and proliferation. In this study, we first explored the impact of miR-148b-3p on POI and found that overexpression of miR-148b-3p reversed the promotion of autophagy and proliferation induced by HOTAIR overexpression. The inhibitory effect of miR-148b-3p inhibitor on KGN cell autophagy and proliferation improvement could also be reversed by silencing ATG14. Overall, our findings indicate the promoting role of HOTAIR in POI and its potential as a biomarker for POI by modulating the miR-148b-3p/ATG14 axis to improve mechanisms of autophagy and proliferation in POI.

## Introduction

Premature ovarian insufficiency (POI), previously referred to as premature ovarian failure, characterizes the premature cessation of typical ovarian function in women below the age of 40 [1, 2]. Roughly 1% of females in their reproductive years are affected by this condition, marked by the early depletion or arrested development of ovarian follicles [3]. Its origins are multifaceted, encompassing idiopathic, genetic, autoimmune and post-chemotherapy factors as common causes [4–7]. However, the underlying factors of nearly half of all cases remain enigmatic, suggesting the involvement of yet-to-be-identified elements in POI progression.

Long non-coding RNAs (lncRNAs), a class of RNA molecules characterized by their length exceeding 200 nucleotides, display substantial diversity and are ubiquitously distributed in both the cell nucleus and cytoplasm [8]. Prior investigations have implicated lncRNAs in the pathogenesis of diverse conditions, including cancer [9], immune dysfunction [10] and embryogenesis [11]. Our previous research also identified aberrantly expressed lncRNAs in POI [12]. Consequently, we are intrigued by the exploration of lncRNAs’ roles in POI. HOTAIR, a lncRNA spanning 2,158 base pairs, originates from the antisense strand of the homeobox C gene locus situated on chromosome 12 and has been linked to aberrations in numerous pathological conditions [13–15]. Despite this, its involvement in POI remains inadequately studied, thus rendering it a focus of our investigation.

Autophagy, a cellular process encompassing the degradation and recycling of damaged cellular components, such as organelles and proteins, maintains cellular homeostasis under normal conditions [16–20]. However, dysregulated autophagy has been implicated in diverse pathological conditions [21]. In the ovarian context, autophagy assumes a pivotal role in selecting dominant follicles and orchestrating follicular atresia [22]. Although some studies have suggested autophagy’s involvement in POI development and as a modifiable therapeutic target [23], the involvement of long non-coding RNAs (lncRNAs) in modulating autophagy remains largely uncharted territory. Considering the fundamental significance of both lncRNAs and autophagy, it becomes imperative to delve into the plausible contributions of lncRNAs to the regulation of autophagic processes.

Limited reports exist regarding the targets of HOTAIR and its impact on granulosa cell progression in POI. In our study, we have demonstrated decreased levels of HOTAIR and increased levels of miR-148b-3p in both POI ovarian tissue and granulosa cells. Furthermore, a comprehensive set of experiments has unveiled that HOTAIR effectively orchestrates the miR-148b-3p/ATG14 axis within the context of POI, resulting in amplified autophagic activity and bolstered cellular proliferation. This groundbreaking study, notably, marks the inaugural establishment of the regulatory framework encompassing HOTAIR/miR-148b-3p/ATG14 in the context of POI. These findings hold significant promise as a novel therapeutic target for individuals grappling with the challenges of POI.

## Result

### The expression level of HOTAIR is decreased in POI, accompanied by reduced levels of autophagy and proliferation

**Fig. 1:**
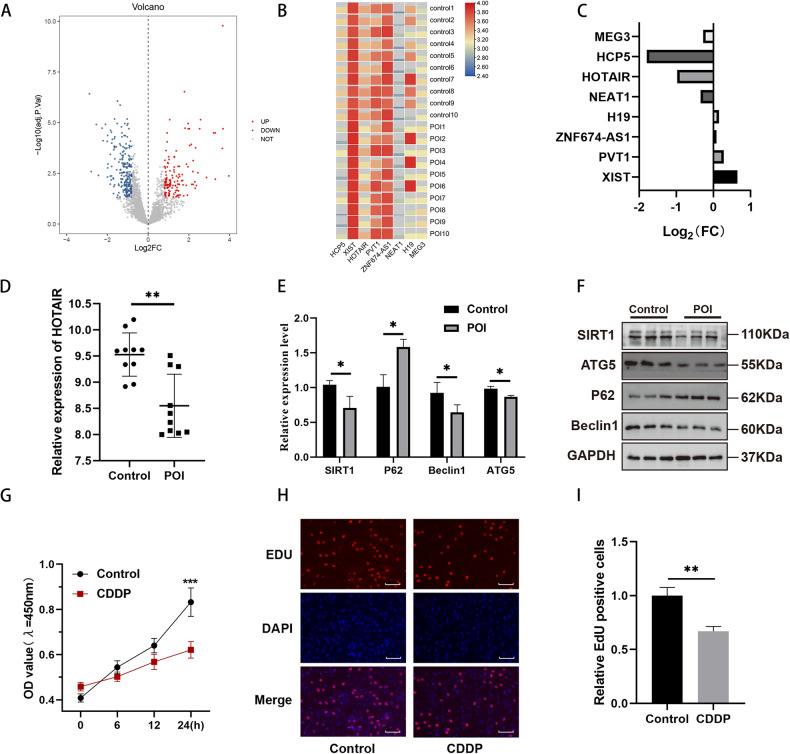
LncRNA HOTAIR is significantly downregulated in CDDP-induced POI and associated with autophagy and proliferation phenomena. **A** Volcano plot depicting extent of upregulated and downregulated lncRNAs along with fold change and p-value cut-offs. **B** Heatmap depicting the expression levels of lncRNAs that have been studied or reported in POI. **C** Distribution of fold change corresponding to the lncRNAs in the heatmap. **D** Quantitative analysis showed a lower level of HOTAIR in the GCs of POI patients (*n* = 10) compared to controls (*n* = 10). Data are presented as mean ± SD. **E** The qRT-PCR analysis of the autophagy gene SIRT1, ATG5, Beclin1 and P62 expression levels in POI mice ovaries. **F** The expression of SIRT1, ATG5, Beclin1 and P62 proteins from control and POI mice was detected by Western blot. **G** The viability of KGN cells after CDDP-induced was assessed by CCK8 assay. Results are expressed as the mean ± SD (*n* = 5). **H**, **I** The proliferation of KGN cells after CDDP was evaluated by EdU staining assay. Results are expressed as the mean ± SD (*n* = 3). Scale bar = 20 μm. *P < 0.05, **P < 0.01, ***P < 0.001.

In our previous study, we observed significant expression differences in many long non-coding RNAs (lncRNAs) when comparing them with normal controls in POI disease [12]. First, by analyzing the GSE135697 dataset with a threshold of log_2_|FC | > 1 and adj.P < 0.05, we identified 93 upregulated lncRNAs and 151 downregulated lncRNAs, as shown in the volcano plot (Fig. 1A). We selected lncRNAs that have been previously reported in ovarian diseases and created a heatmap using TBtools (Fig. 1B). Corresponding to the changes in fold change for these lncRNAs in the above figure (Fig. 1C), we observed that HOTAIR exhibited a negative fold change, similar to the reported results [24]. Subsequently, we specifically highlighted the expression of HOTAIR and found that it was downregulated in POI (Fig. 1D), consistent with the findings of other studies [25].

Following this, we examined the RNA expression levels of autophagy-related genes in ovarian tissues from CDDP-induced mouse models. SIRT1, a member of the Sirtuins family primarily located in the cell nucleus and closely related to autophagy regulation [26, 27], exhibited decreased expression in the POI model. ATG5 and Beclin1 showed a similar trend, while the autophagy-specific substrate P62/SQSTM1 displayed an increasing trend, as shown in Fig. 1E. We also assessed the protein expression levels of ATG5, Beclin1, P62/SQSTM1 and SIRT1, which corroborated the RNA results (Fig. 1F).

Next, we modelled KGN cells with CDDP (10 μM, 24 h) to simulate an in vitro cell model. CCK-8 experiments demonstrated that as the duration of CDDP exposure increased, the absorbance of cells gradually decreased, reaching its maximum difference at 24 h (Fig. 1G). This indicates a time-dependent relationship in CDDP-induced cell models and suggests that CDDP reduces the proliferation of KGN cells. Furthermore, EDU experiments revealed a decrease in the percentage of EDU-positive cells after cisplatin treatment, indicating reduced proliferation levels in the in vitro cell model (Fig. 1H, I). All these observations collectively confirm the presence of inhibited autophagy and decreased proliferation in POI.

### Overexpression of HOTAIR improves proliferation and autophagy levels in the POI model of KGN cells

**Fig. 2:**
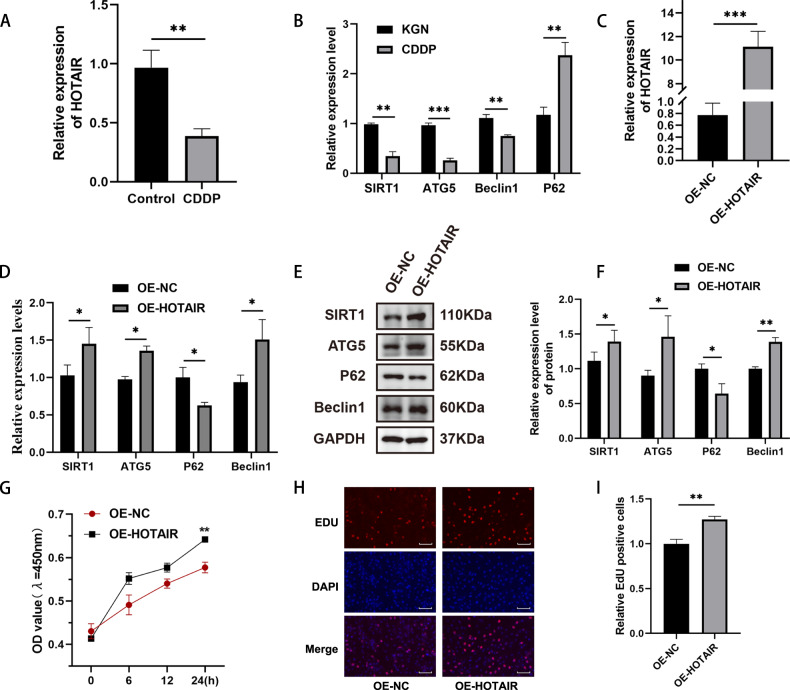
Effects of lncRNA HOTAIR overexpression on the biological functions of CDDP-induced KGN. **A** The qRT-PCR analysis of the lncRNA HOTAIR in CDDP-induced KGN model. **B** The qRT-PCR analysis of the autophagy gene SIRT1, ATG5, Beclin1 and P62 expression levels in the in vitro experiment. **C** Cells transfected with pEX-HOTAIR plasmid showed markedly upregulated the levels of HOTAIR in KGN cells. **D** RNA expression levels of SIRT1, ATG5, Beclin1 and P62 were determined at 24 h after HOTAIR overexpression. **E**, **F** Protein levels of SIRT1, ATG5, Beclin1 and P62 were determined at 24 h after HOTAIR overexpression. **G** Cell viability was measured using CCK-8 assay after HOTAIR overexpression. **H**, **I** The proliferation of KGN cells after HOTAIR overexpression was evaluated by EdU staining assay. Results are expressed as the mean ± SD (*n* = 3). Scale bar = 20 μm. *P < 0.05, **P < 0.01, ***P < 0.001.

In the KGN cell model, we initially examined the expression level of HOTAIR. The RT-qPCR assay revealed a decrease in HOTAIR expression in KGN cell models compared to the normal group (Fig. 2A). Furthermore, consistent results were obtained regarding autophagy levels, validating the stability of the CDDP-model, as observed in the in vitro experiment (Fig. 2B).

To further elucidate the function of HOTAIR in POI, we constructed an overexpression plasmid for lncRNA HOTAIR (pEX-HOTAIR) and analyzed its overexpression efficiency in KGN cells through qRT-PCR (Fig. 2C). In order to investigate the function of HOTAIR, we overexpressed lncRNA HOTAIR in the CDDP-induced cell model. In contrast to the oe-NC group, the overexpression of lncRNA HOTAIR resulted in a significant upregulation of the expression levels of autophagy-related molecules (ATG5, Beclin1, SIRT1), while markedly reducing the levels of P62/SQSTM1 (Fig. 2D). Additionally, the protein levels of ATG5, Beclin1 and SIRT1 were elevated in CDDP-induced KGN cells treated with oe- HOTAIR, while P62/SQSTM1 levels were decreased (Fig. 2E, F).

Moreover, we assessed cell viability using CCK-8 after various transfection conditions(Fig. 2G). The results indicated that, compared to oe-NC, oe-HOTAIR significantly enhanced cell viability and the percentage of EDU-positive cells (Fig. 2H, I). Therefore, we propose that lncRNA HOTAIR might promote KGN autophagy and viability.

### LncRNA HOTAIR can sequester miR-148b-3p through molecular sponging

**Fig. 3:**
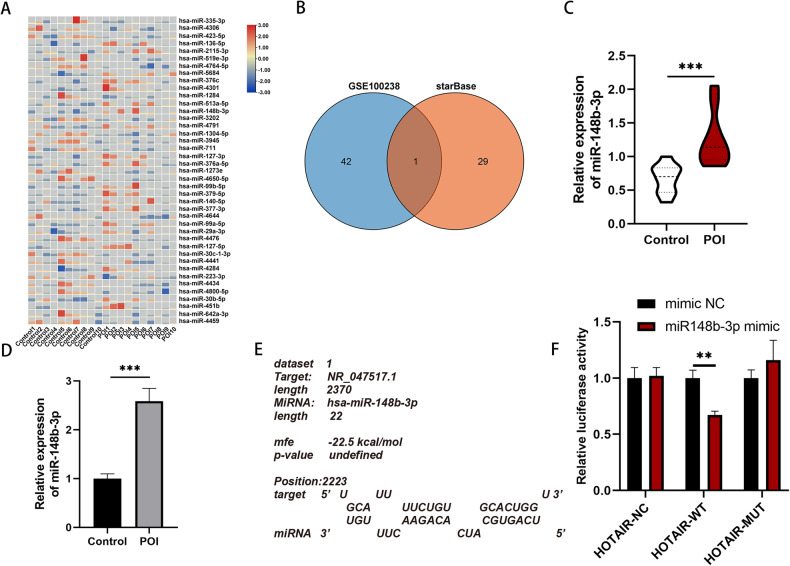
miR-148b-3p is a downstream binding target gene of HOTAIR. **A** Heatmap of miRNAs differentially expressed in POI and control based on the microarray analysis. **B** Venn intersection of GSE100238 and starBase database. **C** Quantitative analysis showed a higher level of miR-148b-3p in the GCs of POI patients (*n* = 10), compared to controls (*n* = 10). Data are presented as mean ± SD. **D** The expression level of miR-148b-3p was validated by qRT-PCR in POI mice. Ct values were normalized to U6. **E** miR-148b-3p predicted to target the HOTAIR using RNAHybrid prediction algorithm. **F** The results of Luciferase reporter gene assays in KGN cells. *P < 0.05, **P < 0.01, ***P < 0.001.

As previously mentioned, the overexpression of HOTAIR enhances the biological behaviours of POI. However, the downstream mechanism of HOTAIR has remained elusive. In an effort to further elucidate the mechanism of HOTAIR in POI patients, downstream target genes of HOTAIR were predicted using the StarBase databases, resulting in 30 candidate downstream molecules. Furthermore, we retrieved microRNA dataset related to POI from the GSE100238 dataset. Differential analysis was conducted using the “Limma” package in the R, with criteria set at log_2_ | FC | > 0.208 and adj.P < 0.05 for screening. In samples from the POI model, 21 significantly upregulated and 21 significantly downregulated miRNAs were identified. A heatmap displaying all upregulated and downregulated miRNAs is shown in Fig. 3A. Subsequently, the Venn diagram displays the intersection from Starbase were compared with miRNAs that exhibited significant changes in the GSE100238 dataset (Fig. 3B), ultimately revealing the unique intersection gene, miR-148b-3p. The expression level of miR-148b-3p in the GSE100238 dataset is depicted in Fig. 3C. We then validated the expression level of miRNA-148b-3p in the POI mouse model, where it showed a significant increase (Fig. 3D). A schematic illustrating the binding sites of miR-148b-3p on lncRNA HOTAIR in humans was generated using RNAhybrid (Fig. 3E).

Utilizing this sequence information, we generated a reporter vector containing both the HOTAIR wild-type (HOTAIR-WT) plasmid and the HOTAIR binding site mutation (HOTAIR-MUT) plasmid for subsequent dual-luciferase reporter assays. The outcomes of these assays revealed a significant decrease in luciferase activity for the HOTAIR-WT upon transfection with the miR-148b-3p mimic. In contrast, there was no discernible impact on the luciferase activity of the HOTAIR-MUT. In comparison to the mimic NC group, the luciferase signal of HOTAIR-WT in the miR-148b-3p mimic group exhibited a substantial decrease, providing compelling evidence to support the specific binding of miR-148b-3p to HOTAIR (Fig. 3F). These collective findings strongly suggest that lncRNA HOTAIR can act as a sequestering agent, binding to and inhibiting miR-148b-3p.

### The miR-148b-3p mimic reversed the regulatory effects of lncRNA HOTAIR overexpression on CDDP-induced biological functions in KGN cells

To delve deeper into the question of whether lncRNA HOTAIR modulates the advancement of POI by acting as a sponge for miR-148b-3p, we conducted functional rescue experiments. These experiments were designed to elucidate whether HOTAIR exerts its effects by affecting miR-148b-3p during the progression of POI.

**Fig. 4:**
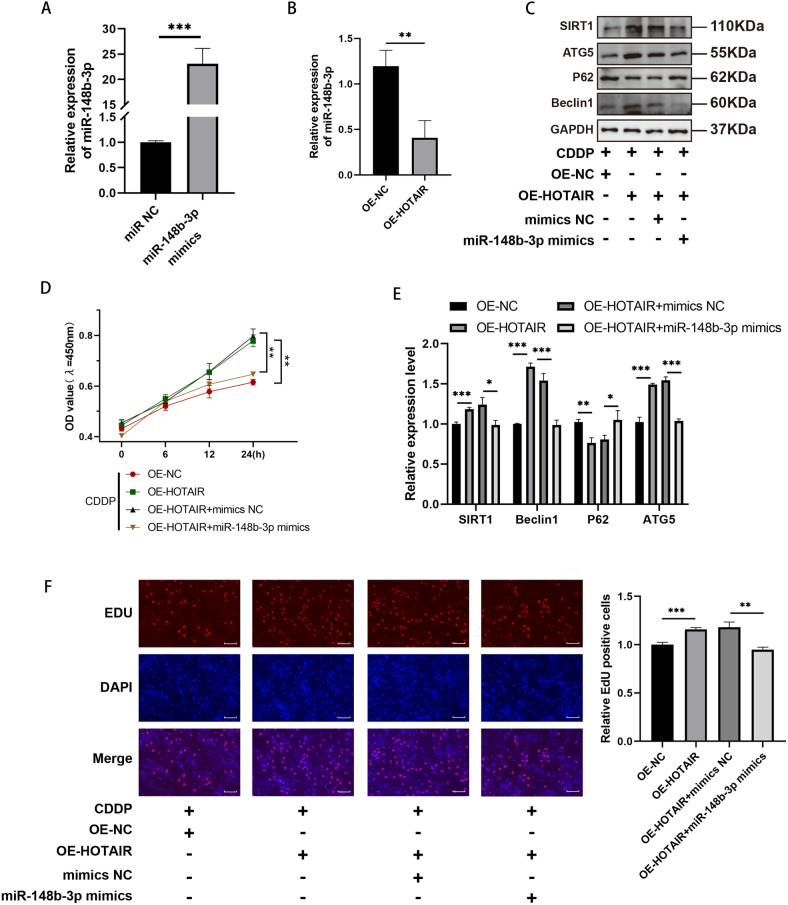
Effects of lncRNA HOTAIR overexpression and miR-148b-3p mimics on the biological functions of CDDP-induced KGN cells. **A** Overexpression efficiency of miR-148b-3p was assessed via qRT-PCR. **B** The expression level of miR-148b-3p was validated by qRT-PCR in overexpression HOTAIR. **C** The western blot of the autophagy gene SIRT1、ATG5、Beclin1 and P62 expression levels in KGN cells with the oe-HOTAIR and miR-148b-3p mimics. **D** CCK-8 assays were used after co-transfection KGN cells with the oe-HOTAIR and miR-148b-3p mimics. **E** The levels of autophagy-related proteins (SIRT1, ATG5, Beclin1 and P62) were detected using qRT-PCR. **F** EdU staining assay showing the proliferation ability of oe-HOTAIR and miR-148b-3p mimics in KGN cells. Results are expressed as the mean ± SD (*n* = 3). Scale bar = 20 μm. *P < 0.05, **P < 0.01, ***P < 0.001.

After confirming that the miR-148b-3p mimics effectively increased miR-148b-3p expression in KGN cells (Fig. 4A), we conducted co-transfections of oe-HOTAIR and the miR-148b-3p mimic into KGN cells. qRT-PCR results clearly demonstrated that the miR-148b-3p expression had been suppressed by the overexpression of oe-HOTAIR (Fig. 4B). Subsequently, in order to further elucidate the role of miR-148b-3p in POI, we performed HOTAIR overexpression and then transfected the cells with the miR-148b-3p mimic. Through qRT-PCR analysis, we found that HOTAIR overexpression led to the suppression of P62/SQSTM1 levels and enhancement of Beclin1, ATG5 and SIRT1 levels, indicating autophagy dysfunction, consistent with the earlier observations. Furthermore, Western blot experiments at the protein expression level confirmed the downregulation of P62/SQSTM1 and the upregulation in Beclin1, ATG5 and SIRT1 levels. However, this phenomenon was reversed after co-treatment with HOTAIR overexpression and the miR-148b-3p mimic, resulting in reduced autophagy as evidenced by decreased Beclin1, ATG5, SIRT1 and increased P62/SQSTM1 (Fig. 4E). Similarly, we observed consistent findings at the protein expression level (Fig. 4C). Finally, concerning proliferation, after treating transfected KGN cells with CDDP in the HOTAIR overexpression group, both cell viability and the proportion of EDU-positive cells exhibited a notable increase compared to the control group. However, in the HOTAIR overexpression and miR-148b-3p mimic co-treatment group, this trend was reversed, manifesting as decreased absorbance and a reduced percentage of EDU-positive cells (Fig. 4D, F).

In conclusion, all the data indicate that HOTAIR’s upregulation of autophagy and promotion of proliferation can be blocked by its downstream molecule, miR-148b-3p, through a sponge mechanism.

### Bioinformatics analysis identified ATG14 as a major downstream target gene of miR-148b-3p

**Fig. 5:**
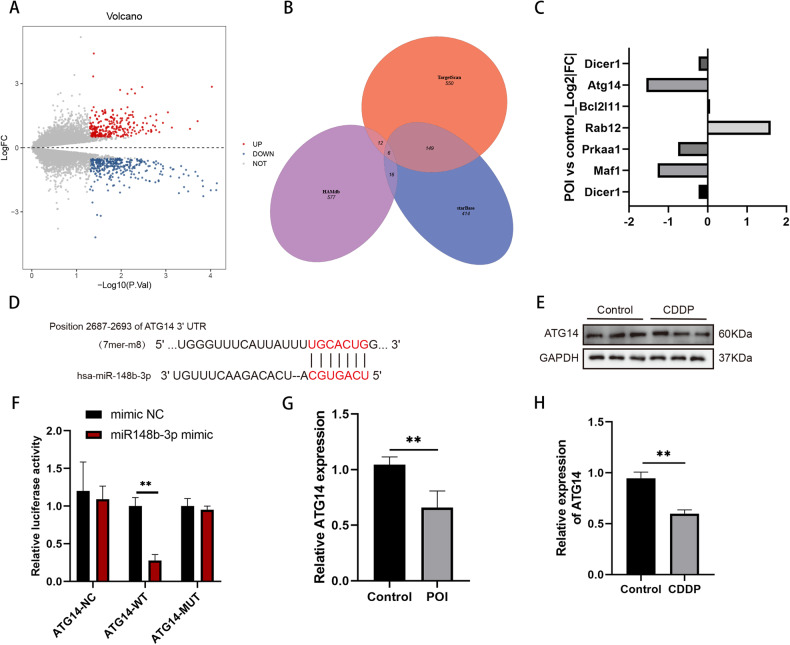
ATG14 competitively sponges miR-148b-3p. **A** The volcano plot of GSE128240 showing downregulated (blue) and upregulated (red) differentially expressed genes. **B** Venn intersection of putative target genes of hsa‐miR-148b-3p based upon three databases (Targetscan, starBase and HAMdb). **C** The log2 fold change of intersecting genes in the dataset. **D** StarBase database predicted the binding sites of miR-148b-3p and ATG14. **E** Protein levels of ATG14 were determined at CDDP-induced KGN cells. **F** Luciferase activities were measured at 48 h post transfection in KGN cells co-transfected with ATG14-Wt or ATG14-Mut reporter and miR-148b-3p mimic or miR-NC. The expression level of ATG14 was validated by qRT-PCR in POI mice (**G**) and CDDP-induced KGN cells (**H**). *P < 0.05, **P < 0.01, ***P < 0.001.

To identify the downstream targets regulated by HOTAIR/miR-148b-3p, we predicted potential target genes of miR-148b-3p through several databases, including StarBase, TargetScan and HAMdb, then identified a total of six common downstream genes (Fig. 5B). Subsequently, we downloaded the relevant geneset GSE128240 from the GEO database and performed differential analysis using the limma package. We set the threshold at log_2_ | FC | > 0.5 and adj.P < 0.05, resulting in 265 upregulated genes and 346 downregulated genes, as depicted in the volcano plot, with red indicating upregulated genes and blue indicating downregulated genes (Fig. 5A). Among the six intersecting genes, ATG14 displayed the largest absolute change in the GSE128240 dataset (Fig. 5C). Hence, we selected ATG14 as the focus of our next research.

To confirm ATG14 as a target of miR-148b-3p, we employed the StarBase 3.0 database and identified a potential binding site between the 3’UTR of ATG14 and miR-148b-3p (Fig. 5D). Dual-luciferase reporter gene assay results demonstrated that the luciferase activity of the wild-type ATG14 3’UTR vector was significantly reduced upon transfection with the miR-148b-3p mimic. In contrast, the luciferase activity of the mutant ATG14 3’UTR vector remained unaffected by the miR-148b-3p mimic. (Fig. 5F). In both the POI mouse model and CDDP-KGN cell model, we assessed the expression level of ATG14 and observed a consistent downregulation (Fig. 5G, H). Furthermore, at the protein level in the cell model, a similar decrease in expression was observed (Fig. 5E), further supporting our conclusion. In summary, these findings indicate that ATG14 is a direct target of miR-148b-3p.

### ATG14 knockdown reversed the regulation of miR-148b-3p on the autophagy and proliferation of CDDP‑induced KGN model

**Fig. 6:**
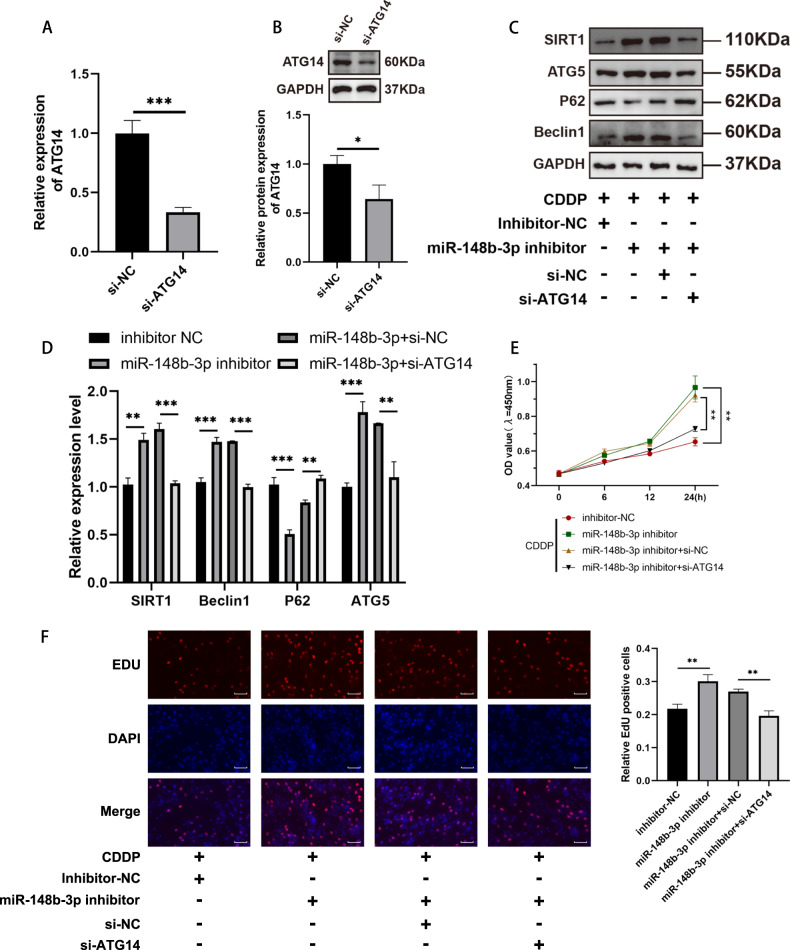
Effects of miR-148b-3p and ATG14 on the autophagy and proliferation of CDDP-induced KGN cells. **A** The qRT-PCR showing knockdown of ATG14 in KGN cells. **B** Western blot analysis confirmed a marked reduction of ATG14 protein following ATG14 small interfering RNA transfection in KGN cells. **C** The levels of autophagy-related proteins (SIRT1, ATG5, Beclin1 and P62) were detected using western blotting. **D** The qRT-PCR analysis of the autophagy gene SIRT1、ATG5、Beclin1 and P62 expression levels in KGN cells with the miR-148b-3p inhibitor and si-ATG14. **E** CCK-8 assays were used after co-transfection KGN cells with the miR-148b-3p inhibitor and si-ATG14. **F** EdU staining assay showing the proliferation ability of miR-148b-3p inhibitor and si-ATG14 in KGN cells. Results are expressed as the mean ± SD (*n* = 3). Scale bar = 20 μm. *P < 0.05, **P < 0.01, ***P < 0.001.

To delve deeper into whether ATG14 governs the advancement of POI by sequestering miR-148b-3p, we performed functional rescue experiments to elucidate if ATG14 contributes to the progression of POI by modulating miR-148b-3p. We synthesized siATG14 and its negative control siNC and transfected them into KGN cells, confirming that siATG14 achieved the intended knockdown at the RNA level (Fig. 6A). Subsequently, we validated this at the protein level, demonstrating successful knockdown of ATG14 gene/protein expression through small RNA interference (Fig. 6B).

Next, to further elucidate the role of ATG14 in POI, we inhibited miR-148b-3p with si-ATG14 transfection. Through qRT-PCR analysis, we found that after inhibiting miR-148b-3p, P62/SQSTM1 levels decreased, while Beclin1, ATG5 and SIRT1 levels increased, indicating an improvement in autophagic function compared to the control group (Fig. 6D). Western blot analysis of protein expression levels confirmed the downregulation of P62/SQSTM1 and the upregulation of Beclin1, ATG5 and SIRT1, further validating our hypothesis. However, after the combined treatment of inhibiting miR-148b-3p and si-ATG14, this phenomenon was reversed, resulting in decreased autophagy, characterized by a reduction in Beclin1, ATG5 and SIRT1 and an increase in P62/SQSTM1 levels (Fig. 6C).

Finally, concerning cell proliferation, CDDP treatment notably boosted cell viability and the ratio of EDU-positive cells in KGN cells transfected with miR-148b-3p inhibitor. However, in the group subjected to both miR-148b-3p inhibitor and si-ATG14 treatment, this trend reversed, resulting in decreased absorbance and a reduced percentage of EDU-positive cells. (Fig. 6E, F).

In summary, all the data suggest that the upregulation of autophagy and promotion of proliferation by inhibiting miR-148b-3p can be counteracted by its downstream molecule, ATG14, through a sponge mechanism.

## Discussion

**Fig. 7:**
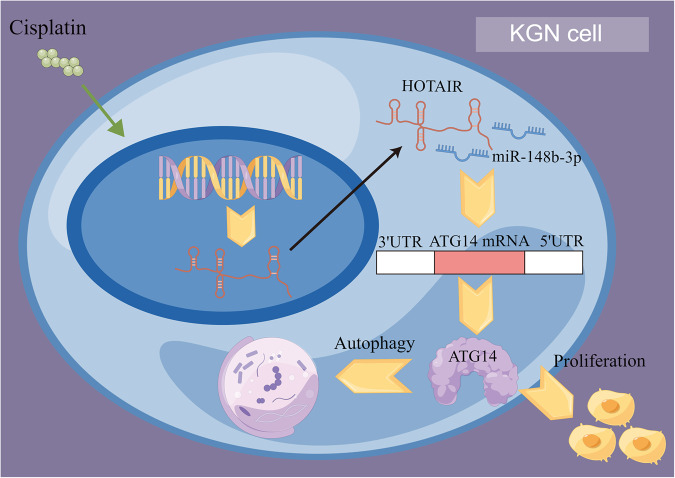
The mechanism of HOTAIR regulating autophagy and proliferation in POI. In the presence of cisplatin (CDDP), long non-coding RNA HOTAIR regulates the miR-148b-3p/ATG14 axis, thereby promoting autophagy and proliferation in human ovarian granulosa cells (KGN), as depicted in FigDraw.

The results of this study suggest a decreased expression of lncRNA HOTAIR in POI patients, accompanied by reduced levels of autophagy. Under normal conditions, adequate levels of lncRNA HOTAIR can regulate autophagy via its interaction with miR-148b-3p, ensuring an appropriate level of ATG14 protein to maintain autophagy and promote the proliferation capacity of granulosa cells (Fig. 7).

The mechanistic role of HOTAIR has been found to be crucial in developing various ovarian conditions, such as polycystic ovary syndrome [28], ovarian cancer [29], and endometriosis [30]. Additionally, HOTAIR may impact the outcome of chemotherapy by modulating autophagy in conditions like endometrial carcinoma [31] and non-small cell lung cancer [32]. In this study, the microarray results demonstrated decreased expression of HOTAIR in POI. Furthermore, by simulating post-chemotherapy conditions in female mice through intraperitoneal injection of CDDP, we confirmed a significant reduction in HOTAIR expression in ovarian samples from POI patients after chemotherapy, consistent with previous research findings [33]. Similarly, in in vitro experiments, we observed a decrease in lncRNA HOTAIR levels in KGN cells after CDDP treatment. Phenotypically, our study indicates that decreased levels of autophagy and proliferation accompany the reduction in HOTAIR expression. Bao et al. [34] provided evidence of HOTAIR overexpression triggering elevated autophagic activity, improving cell viability and decreasing apoptosis rates in chondrosarcoma. This regulatory effect was attributed to HOTAIR’s modulation of the miR-454-3p/ATG12 pathway. Therefore, we further investigated whether HOTAIR could regulate proliferation by modulating autophagy in POI. Overexpression of HOTAIR enhanced autophagy levels in KGN cells.

Additionally, reports have suggested that HOTAIR promotes the proliferation of breast cancer CSCs and influences stem cell proliferation and differentiation [35, 36]. Our results similarly indicate that overexpression of HOTAIR in vitro promotes proliferation. These data collectively suggest that HOTAIR may impact POI by upregulating autophagy and promoting proliferation.

MiR-148b-3p is a member of the miR-148/miR-152 family, which encompasses miR-148a and miR-152 as well [37]. miR-148b-3p has been found to be closely associated with the onset and progression of certain diseases, such as schizophrenia [38], and the prognosis of ovarian tumors [39]. Furthermore, previous research has indicated that miR-148b-3p regulates pancreatic autophagy by inhibiting autophagy-related protein 12 (ATG12) [40]. However, the role of miR-148b-3p in autophagy in the context of POI remains largely unknown. It has been reported that autophagy is involved in regulating granulosa cell (GCs) apoptosis, accelerating follicular atresia [41]. Nevertheless, the potential mechanisms of autophagy dysfunction in GCs’ functional impairment are yet to be determined. Nevertheless, autophagy has become a crucial mechanism for maintaining internal balance, particularly in terms of ovarian function and female reproduction [42].

The initiation of autophagy, representing the first step, depends on the formation of the Class III phosphatidylinositol 3-kinase complex, which consists of PIK3C3/Vps34 (mammalian/yeast protein), PIK3R4/Vps15, BECN1/Vps30/Atg6 and ATG14/atg14 [43]. Recent reports indicate a substantial reduction in ATG14 levels within the endometrium of individuals diagnosed with polycystic ovary syndrome [44]. However, no previous studies have investigated this molecule in the context of POI.

Considering the intricate interplay among HOTAIR, miR-148b-3p and ATG14, our investigation delved deeper into whether HOTAIR could exert its regulatory influence in POI by modulating miR-148b-3p and its downstream target, ATG14. Through our study, we have elucidated that HOTAIR suppresses the expression of miR-148b-3p through direct interaction, and ATG14 is confirmed as a direct target of miR-148b-3p. Furthermore, HOTAIR actively promotes the expression of ATG14 in KGN cells, thus substantiating its role as a molecular sponge for miR-148b-3p, ultimately inducing the expression of ATG14 in the context of POI.

Functional analysis has unveiled a significant outcome that overexpression of HOTAIR results in the downregulation of miR-148b-3p. This downregulation, in turn, is associated with heightened cell viability and an upswing in autophagic activity within KGN cells. However, this effect was significantly attenuated when miR-148b-3p mimic was used, suggesting that high expression of miR-148b-3p enhances cisplatin sensitivity in KGN cells, resulting in reduced levels of autophagy and proliferation. Furthermore, our study demonstrated a significant downregulation of ATG14 protein expression in POI. Additionally, in POI, ATG14 protein expression showed a negative correlation with miR-148b-3p expression and a positive correlation with HOTAIR expression. By elevating ATG14 using a miR-148b-3p inhibitor, we observed increased cell viability and autophagic activity in KGN cells. However, this effect was markedly diminished when si-ATG14 was used, indicating that ATG14 deficiency enhances cisplatin sensitivity in KGN cells, resulting in decreased levels of proliferation.

In conclusion, our findings strongly indicate that HOTAIR plays a pivotal role in promoting proliferation by modulating the miR-148b-3p/ATG14-mediated autophagy pathway. This study provides comprehensive insights into the intricate mechanisms through which HOTAIR regulates autophagy and proliferation in the context of POI. Furthermore, our results underscore the potential therapeutic significance of HOTAIR in mitigating, treating, and preventing cisplatin-induced toxicity in POI. Nevertheless, it is imperative to further substantiate our conclusions by employing autophagy modulators in both in vitro and in vivo settings.

## Materials and methods

### Animals

Female ICR mice, meeting the specific-pathogen-free (SPF) grade criteria and aged between 7 and 8 weeks, were procured from Zhaoyan Laboratory Animal (Suzhou, China). These mice were housed in a controlled laboratory environment characterized by a temperature range of 22–23 °C, humidity levels maintained at 55–60%, and a standard 12 h light/dark cycle. Before the experiment, the mice were randomly divided into control group and intervention group. The Cisplatin-induced premature ovarian insufficiency (CDDP-POI) model was constructed according to the previous study [45]. Female mice were weighed and then given a 7-day intraperitoneal injection of cisplatin (2 mg/kg) or an equal volume of saline solution as a control. After one week, the mice were euthanized, and ovarian tissue was collected for further study.

### Bioinformatics analysis

We utilized data from the Gene Expression Omnibus (GEO) database, specifically identified under accession number GSE135697, to discern variations in the expression of long non-coding RNAs (lncRNAs), encompassing 10 cases of patients with POI and 10 control samples. The construction of the volcano plot was conducted employing the R package “ggplot2”. Subsequently, the heatmap visualization was accomplished using TBtools [46].

In parallel, differential expression of microRNAs was ascertained through analysis of the GSE100238 dataset, which features 10 POI cases and ten normal controls. The prediction of target genes for HOTAIR was achieved utilizing the starBase databases. The resultant intersection of microRNAs was depicted using TBtools. In addition, we conducted predictions of downstream target genes regulated by miR-148b-3p using comprehensive resources, including starBase and the GSE128240 dataset. The creation of Venn diagrams was executed employing the R package “eulerr”, with the size of intersected regions reflective of the count of overlapping genes.

### Cell culture, treatment and transfection

KGN cells were kindly donated by Dr Li Chen (Chinese PLA General Hospital, Nanjing, China). The cells were cultured in a 5% CO_2_ incubator at 37 °C in Dulbecco’s Modified Eagle’s Medium (DMEM)/F12 medium supplemented with 10% fetal bovine serum (Sigma, USA) and 1% penicillin/streptomycin (Gibco, USA).

For the CDDP-induced POI cell model, KGN was cultured in a complete medium containing CDDP (10 μM, MCE, USA) for 24 h. For cell transfection, KGN was seeded into 6-well plates overnight and up to 50%-70%. The HOTAIR overexpression plasmid (pEX-HOTAIR), miR-148b-3p mimic and inhibitor, small interference RNA of autophagy-related gene 14 (si-ATG14) and corresponding negative controls (pEX-NC, mimic NC, inhibitor NC, and si-NC) were synthesized from GenePharma (Suzhou, China). The corresponding sequences are in supplementary tables 1 and 2. KGN cells were transfected using Lipofectamine 6000 (Beyotime, Shanghai, China) following the manufacturer’s instructions. Subsequently, after 6 h, the culture medium was replaced with a fresh, complete medium.

### Cell counting kit 8 (CCK8) assay

KGN cells were seeded in 96-well petri dishes at a density of 5 × 10^^3^ cells per well and allowed to incubate overnight. The cells were then treated with 10 μM cisplatin for 0, 6, 12 and 24 h. After reaching the designated time points, the culture medium was replaced with 100 μL of DMEM solution containing 10% CCK-8 reagent for measuring cell viability. After incubating for 60 min, the absorbance at 450 nm was measured.

### EDU assay

KGN cells were cultured overnight in 24-well plates, followed by intervention with cisplatin for 24 h, and then cultured under different transfection conditions for 48 h. To assess cell proliferation, the EDU Cell Proliferation Assay Kit (Beyotime, Shanghai, China) was used to stain cells, and DAPI was used for nuclear counterstaining. The quantity of EDU-positive cells was examined utilizing a fluorescence microscope, and the outcomes were assessed by computing the proportion of EDU-positive cells to those stained with DAPI.

### Western blot

Cellular and ovarian lysates were prepared using RIPA lysis buffer (Elabscience, Wuhan, China), with the addition of protease and phosphatase inhibitors. Protein concentrations were quantified using the BCA Protein Concentration Assay Kit (Beyotime, Shanghai, China). Following this, 20 μg of protein was subjected to separation via SDS-PAGE and subsequently transferred onto a polyvinylidene difluoride (PVDF) membrane (Millipore, Boston, USA). The membranes were blocked in 5% skimmed milk for 2 h and probed with antibodies against SIRT1(proteintech, 13161-1-AP, 1:1000), ATG14 (proteintech, 19491-1-AP, 1:1000), Beclin1 (CST, 3495, 1:1000), P62/SQSTM1 (CST, 39749, 1:1000), ATG5 (CST, 12994, 1:1000) and GAPDH (proteintech, 60004-1-Ig, 1:10000) overnight at 4 °C. Subsequently, the membranes were exposed to secondary antibodies for 1 h at room temperature. Protein bands were visualized utilizing the ECL method, and the intensity of the bands was quantified using Image J (NIH, USA).

### RNA extraction, reverse transcription, and quantitative realtime PCR analysis (qRT-PCR)

Total RNA was isolated employing an RNA quick purification kit (Yishan, Shanghai, China), and the RNA integrity was assessed using the Qubit™ RNA HS Assay Kit (Invitrogen, Carlsbad, CA). Complementary DNA (cDNA) was synthesized using the ABScript III Reverse Transcriptase(Abclonal, Suzhou, China). Regarding miRNA, complementary DNA (cDNA) was synthesized in a reverse transcription reaction using stem-loop primers and the miRNA 1st Strand cDNA Synthesis Kit (Vazyme, Nanjing, China). Quantitative real-time polymerase chain reaction (qRT-PCR) was conducted utilizing the SYBR method. The 2^−ΔΔCt^ method was employed to determine relative expression levels, with GAPDH or U6 serving as internal controls. Reference for primers can be found in supplementary table 3.

### Dual‑luciferase reporter assay

To prepare reporter plasmids HOTAIR-WT/ATG14-WT, the sequences containing miR-148b-3p binding sites within HOTAIR and the 3′‐untranslated region (3′‐UTR) of ATG14 were inserted into the pmirGLO vector (Geenpharma, Suzhou, China). Reporter plasmids with mutated sequences, named HOTAIR-MUT/ATG14-MUT, were constructed as negative controls using mutated sequences of HOTAIR/ATG14. The constructed reporter plasmids (500 ng/μL) and miR-148b-3p mimic (20 μM) were transfected into KGN cells using Lipo6000™ transfection reagent (Beyotime, Shanghai, China). Following a 48-hour incubation period, the Dual-Luciferase Reporter Assay System (Promega, USA) was employed to assess the relative activities of firefly and Renilla luciferases. This system facilitated the quantification of luciferase activity and the determination of the relative activities of these two luciferases.

### Statistical analysis

All experiments were independently replicated a minimum of three times. Statistical analyses were performed using GraphPad Prism Software 8.0 (GraphPad, USA). Data are expressed as mean ± standard deviation. Student’s t-test was used to assess differences between two groups, while one-way analysis of variance (ANOVA) was employed to evaluate differences among more than two groups. A significance threshold of *p* < 0.05 was regarded as statistically significant.

### Supplementary information

A reworked supplementary form file

Protein electrophoresis gel image

Protein electrophoresis gel image

## Data Availability

The data used and analyzed during the current study are available from the corresponding author on reasonable request.
