# Supplementary material for: LncRNA HOTAIR regulates autophagy and proliferation mechanisms in premature ovarian insufficiency through the miR-148b-3p/ATG14 axis
Source: Cell Death Discov. 2024 Jan 24;10:44. doi: 10.1038/s41420-024-01811-z (PMC10808186; doi:10.1038/s41420-024-01811-z)
Supplement: Supplementary file 1 — A reworked supplementary form file [file 41420_2024_1811_MOESM1_ESM.docx]

**Supplementary Information**

**LncRNA HOTAIR regulates autophagy and proliferation mechanisms in premature ovarian insufficiency through the miR-148b-3p/ATG14 axis**

Chao Luo1†, Lun Wei1† , Fei Qian1†, Le Bo, Shasha Gao1, Guangzhao Yang1，Caiping Mao1*

Reproductive Medicine Center, First Affiliated Hospital of Soochow University, Suzhou 215000 China.

* Correspondence:

*Corresponding author: Caiping Mao, Reproductive Medicine Center, The First Affiliated Hospital of Soochow University, 899 Pinghai Rd, Suzhou, Jiangsu, China, maocaiping@suda.edu.cn.

†These authors have contributed equally to this work and share first authorship.

**Supplementary Tables**

**Table S1.** Sequences of siRNA.

| **Name** | **Sequence** |
| --- | --- |
| *ATG14* siRNA | 5’-GGGAGAGGUUUAUCGACAATT-3’  5’-UUGUCGAUAAACCUCUCCCTT-3’ |

**Table S2.** Sequences of microRNA mimics.

| **Name** | **Sequence** |
| --- | --- |
| *has-miR-148b-3p-mimics* | 5’-UCAGUGCAUCACAGAACUUUGU-3’  5’-AAAGUUCUGUGAUGCACUGAUU-3’ |

**Table S3.** Primers used in the experiments.

| **Gene** | **Sequence** | |
| --- | --- | --- |
| *hsa-GAPDH* | Forward | 5’- GGAGCGAGATCCCTCCAAAAT-3’ |
|  | Reverse | 5’- GGCTGTTGTCATACTTCTCATGG-3’ |
| *U6* | Forward | 5’- CTCGCTTCGGCAGCACA-3’ |
|  | Reverse | 5’- AACGCTTCACGAATTTGCGT-3’ |
| *hsa-ATG5* | Forward | 5’-TTTGCATCACCTCTGCTTTC-3’ |
|  | Reverse | 5’-TAGGCCAAAGGTTTCAGCTT-3’ |
| *hsa-Beclin1* | Forward | 5’- GAGGGATGGAAGGGTCTA-3’ |
|  | Reverse | 5’- GCCTGGGCTGTGGTAAGT-3’ |
| *hsa-P62* | Forward | 5’- TGTGTAGCGTCTGCGAGGGAAA-3’ |
|  | Reverse | 5’- AGTGTCCGTGTTTCACCTTCCG-3’ |
| *hsa-HOTAIR* | Forward | 5’- CAGTGGGGAACTCTGACTCG-3’ |
|  | Reverse | 5’- GTGCCTGGTGCTCTCTTACC-3’ |
| *hsa-ATG14* | Forward | 5’- CGTCTACTTCGACGGCCGCGA-3’ |
|  | Reverse | 5’- CTCTTGGTGCCGTTGTGCTCG-3’ |
| *hsa-SIRT1* | Forward | 5’- GCTGGCCTAATAGAGTGGCAA-3’ |
|  | Reverse | 5’- CTCAGCGCCATGGAAAATG-3’ |
| *mmu-GAPDH* | Forward | 5’- AGGTCGGTGAACGGATTTG-3’ |
|  | Reverse | 5’- TGTAGACCATGTAGTTGAGGTCA-3’ |
| *mmu-SIRT1* | Forward | 5’- GCTGACGACTTCGACGACG-3’ |
|  | Reverse | 5’- TCGGTCAACAGGAGGTTGTCT-3’ |
| *mmu-HOTAIR* | Forward | 5’-CCTTATAAGCTCATCGGAGCA-3’ |
|  | Reverse | 5’- CATTTCTGGGTGGTTCCTTT-3’ |
| *mmu-P62* | Forward | 5’- AGGATGGGGACTTGGTTGC -3’ |
|  | Reverse | 5’- TCACAGATCACATTGGGGTGC -3’ |
| *mmu-Beclin1* | Forward | 5’- ATGGAGGGGTCTAAGGCGTC -3’ |
|  | Reverse | 5’- TCCTCTCCTGAGTTAGCCTCT -3’ |
| *mmu-ATG5* | Forward | 5’- TGTGCTTCGAGATGTGTGGTT -3’ |
|  | Reverse | 5’- GTCAAATAGCTGACTCTTGGCAA -3’ |
| *mmu-ATG14* | Forward | 5’- GAGGGCCTTTACGTGGCTG -3’ |
|  | Reverse | 5’-AATAGACGAAATCACCGCTCTG -3’ |
| miR-148b-3p-RT | GTCGTATCCAGTGCAGGGTCCGAGGTATTCGCACTGGATACGACACAAAG | |
| miR-148b-3p-F |  | 5’-CGCGTCAGTGCATCACAGAA-3’ |
| mQ Primer R |  | 5’-AGTGCAGGGTCCGAGGTATT-3’ |
