# Supplementary figures and images for: LncRNA HOTAIR regulates autophagy and proliferation mechanisms in premature ovarian insufficiency through the miR-148b-3p/ATG14 axis
Source: Cell Death Discov. 2024 Jan 24;10:44. doi: 10.1038/s41420-024-01811-z (PMC10808186; doi:10.1038/s41420-024-01811-z)

Figure1F

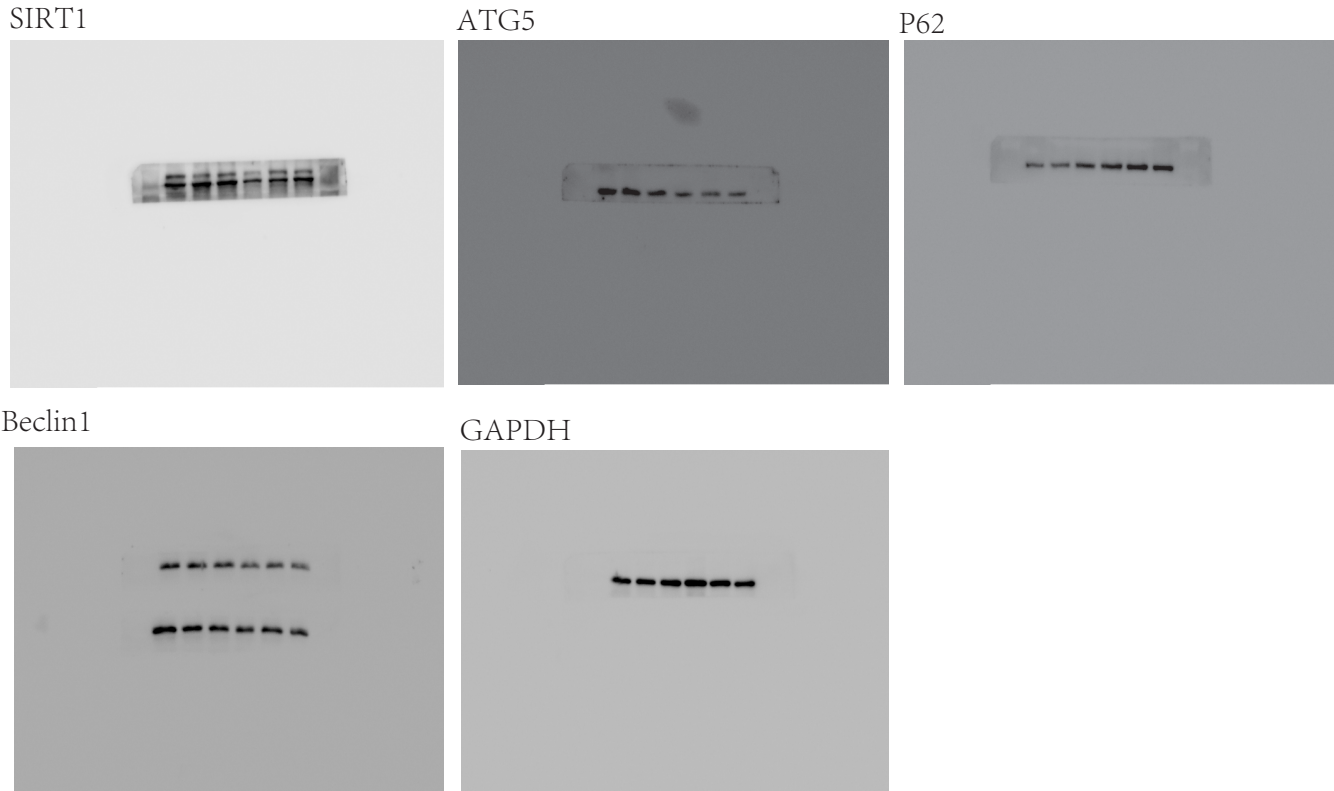

Figure2E

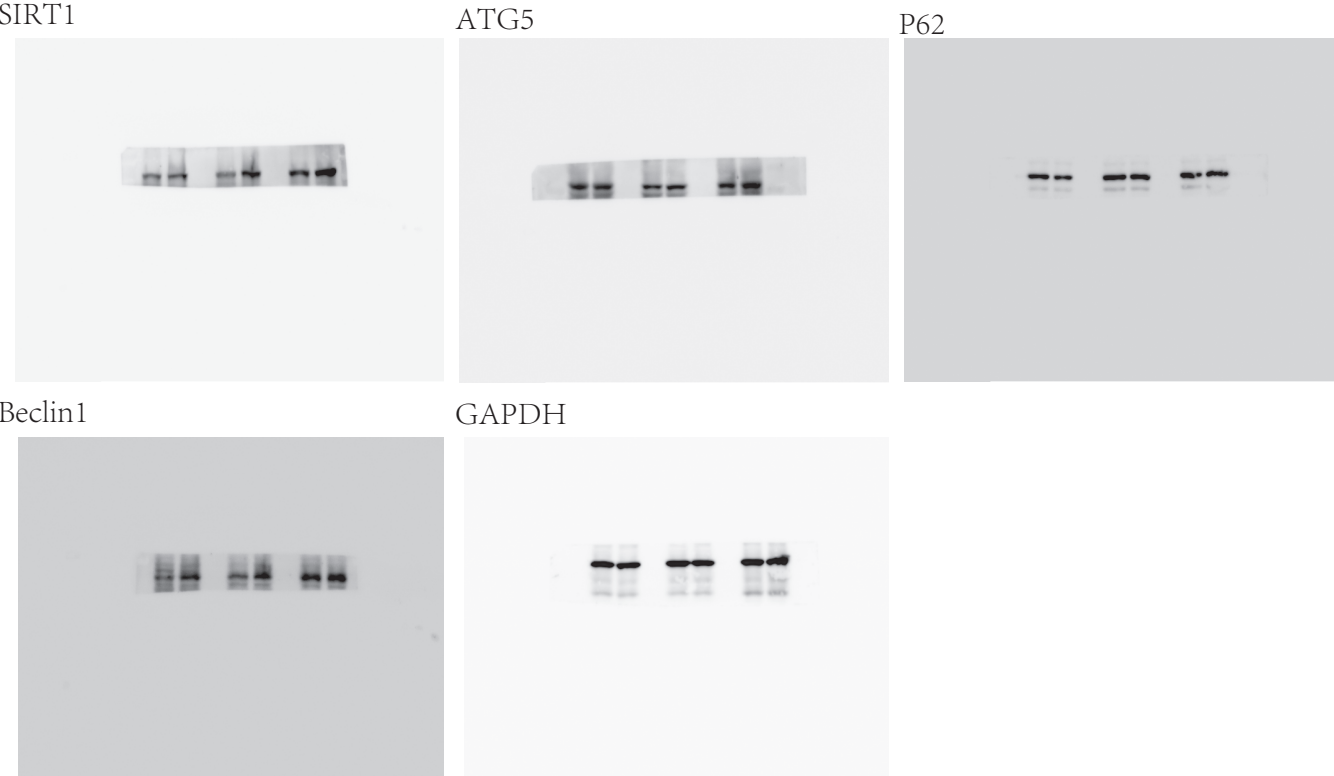

Supplement: Supplementary file 2 — Protein electrophoresis gel image [file 41420_2024_1811_MOESM2_ESM.pdf]

Figure4C

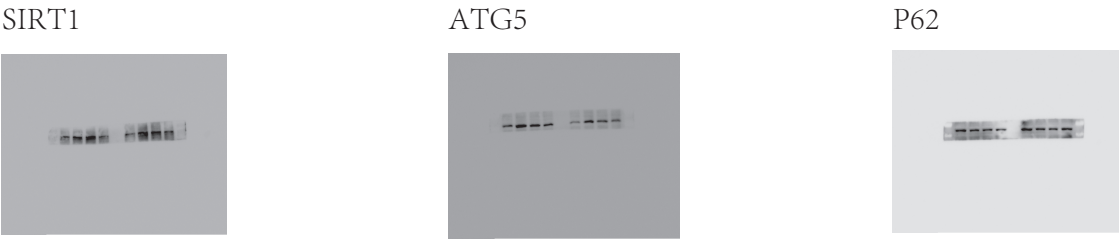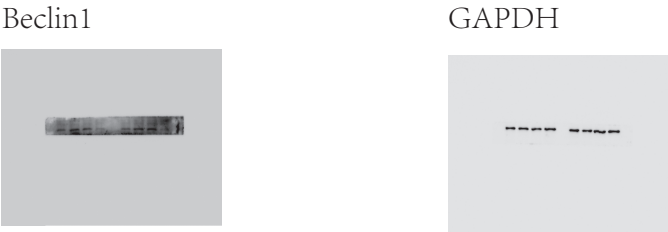

Figure5E

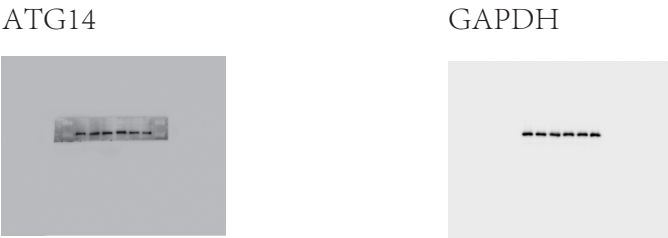

Figure6B

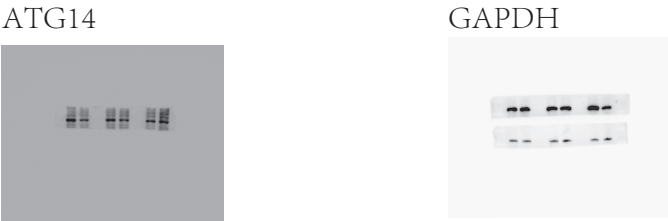

Figure6C

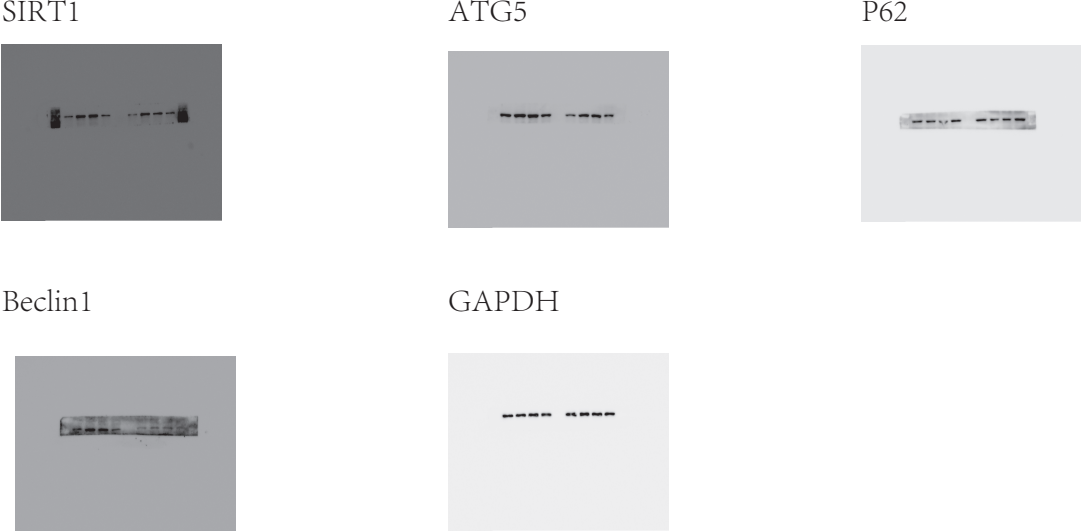

Supplement: Supplementary file 3 — Protein electrophoresis gel image [file 41420_2024_1811_MOESM3_ESM.pdf]
